# Percutaneous transhepatic cholangiography versus endoscopic retrograde cholangiography for the pathological diagnosis of suspected malignant bile duct strictures

**DOI:** 10.1097/MD.0000000000019545

**Published:** 2020-03-13

**Authors:** Hai-Yang Chang, Bin Liu, Yong-Zheng Wang, Wu-Jie Wang, Wei Wang, Dong Li, Yu-Liang Li

**Affiliations:** a Department of Intervention Medicine, the Second Hospital of Shandong University; b Interventional Oncology Institute of Shandong University, Jinan, Shandong Province, People's Republic of China.

**Keywords:** brush cytology, endoscopic retrograde cholangiography, forceps biopsy, malignant bile duct stricture, percutaneous transhepatic cholangiography

## Abstract

To compare the diagnostic performance of percutaneous transhepatic cholangiography and endoscopic retrograde cholangiography for the pathological assessment of suspected malignant bile duct stricture, using brush cytology and forceps biopsy.

The study group comprised 79 consecutive patients who underwent pathological assessment for suspected malignant biliary stricture, 38 of whom underwent percutaneous transhepatic cholangiography (group A) and the other 41 underwent endoscopic retrograde cholangiography (group B). The sensitivity, specificity, positive predictive value, negative predictive value, and accuracy were calculated. A subset analysis was performed to determine the effect of location and pathological type of the stricture on diagnostic performance, and complications were analyzed.

The sensitivity, specificity, positive predictive value, negative predictive value, and accuracy were 86.7%, 100%, 100%, 66.7%, and 89.5%, respectively, in group A, and 77.1%, 100%, 100%, 42.9%, and 80.4%, respectively, in group B. For hilar biliary strictures, the sensitivity and accuracy were superior in group A than in group B. Mild complications (transient c and bile leakage) were identified in 7 cases in each group, all resolved spontaneously within 3 to 5 days.

Both brush cytology and forceps biopsy performed during percutaneous transhepatic cholangiography and endoscopic retrograde cholangiography provided good diagnostic sensitivity and accuracy. Therefore, both diagnostic approaches can play an important role in planning therapeutic strategy. However, for strictures located at the hilum, pathology sampling via percutaneous transhepatic cholangiography is preferable to endoscopic retrograde cholangiography, as it provides higher sensitivity and accuracy.

## 1 Introduction

The therapeutic strategy for biliary stricture remains controversial due to the lack of standardized preoperative histological or cytological diagnostic methods for the differentiation between malignant and benign lesions. Abdominal ultrasound, computed tomography (CT) and magnetic resonance cholangiopancreatography (MRCP) are noninvasive imaging techniques that are used to assess stenosis of the bile duct.^[1]^ Multidetector computed tomography (MDCT) cholangiography, with volume rendering, can further identify a malignant biliary obstruction with high sensitivity and specificity.^[2]^ However, there are still some cases suspected malignant biliary stricture that cannot be accurately diagnosed by imaging alone.

Percutaneous transhepatic cholangiography (PTC) was developed several decades ago to visualize a biliary obstruction and for palliative management of malignant obstructive jaundice.^[3]^ Since the 1980s, percutaneous biliary catheterization has been used to provide an access for brush cytology, fine needle aspiration, and forceps biopsy during PTC.^[4–6]^ More recently, endoscopic retrograde cholangiopancreatography (ERCP) has been accepted as a well-established diagnostic and therapeutic technique for biliary stenosis.

With regard to pathological assessment, brush cytology remains the first-line diagnostic method during ERCP, due to its feasibility and diagnostic specificity, regardless of its low sensitivity.^[7–9]^ However, as intraductal forceps biopsy provides a higher yield of diagnostic sensitivity than brush cytology, it may play an important role in the pathological confirmation of biliary stricture.^[10–11]^ Therefore, the aim of our retrospective study was to compare the diagnostic performance of PTC and ERCP for the pathological assessment of suspected malignant bile duct stricture, using both brush cytology and forceps biopsy.

## 2 Materials and methods

Seventy-nine consecutive patients with a diagnosis of suspected malignant biliary stricture were retrospectively identified from our hospital patient database and enrolled in our study group. Our institutional ethics committee approved our study and the written informed consent was obtained from all patients.

### 2.1 Patients

**Figure 1:**
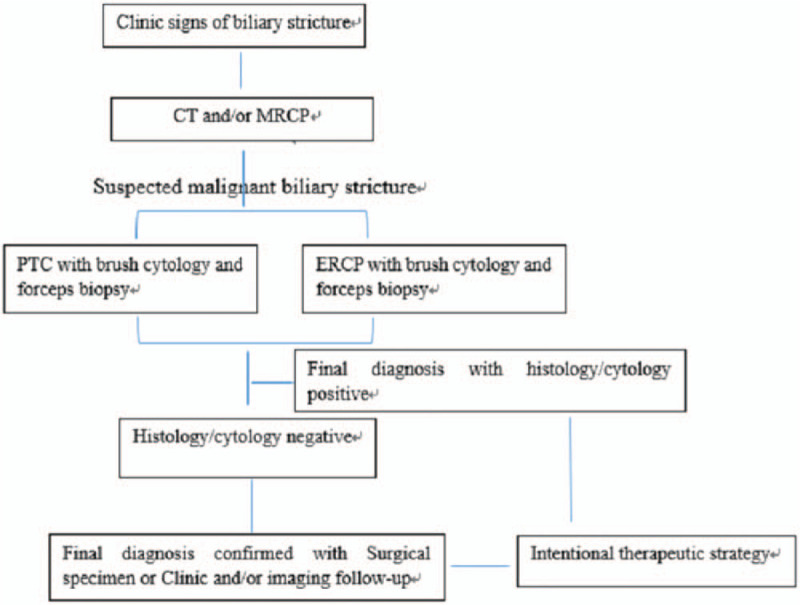
The diagnostic algorithm of suspected malignant biliary stricture.

Relevant baseline characteristics of our study group are summarized in Table 1. All patients presented with jaundice, abdominal discomfort, and/or poor appetite related to biliary stricture. Enhanced CT and/or MRCP imaging was performed for all patients, with suspected malignant biliary stricture being an indication for PTC or ERCP. The diagnostic algorithm applied is shown in Figure 1. Of the 79 patients in our study group, 38 underwent PTC and the other 41 underwent ERCP (with forceps biopsy always performed before brush cytology). The following data were extracted from patients’ records to be included in the analysis: age, sex, site of the lesion, and histology. The procedure was deemed to be successful when a volume of tissue specimen sufficient for diagnosis was obtained. For the purpose of analysis, histological samples were classified as suspected malignancy, atypical, or benign (compared to samples of confirmed malignancy).

**Table 1:**
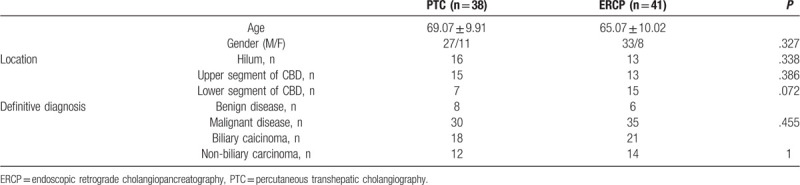
Baseline characteristics of the patients.

### 2.2 Procedures and techniques

Both PTC and ERCP were performed using standard techniques, with brush cytology and forceps biopsy performed in the same session.

**Figure 2:**
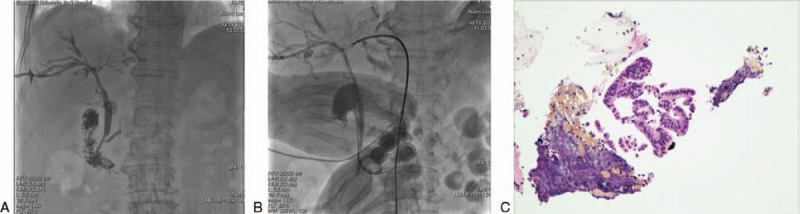
A. cholangiography showed lesion involved the porta hepatis and stricture of right and left hepatic bile duct. 2B. biopsy was performed by forceps deployed in the porta hepatis. 2C. adenocarcinoma was demonstrated by pathology.

For PTC, procedures were performed under intravenous sedation and local anesthesia. After puncture of the bile duct, a sheath was advanced into the biliary tree over the guidewire and cholangiography was performed to show the site, degree and extent of the stricture. A Vert catheter, along with a stiff hydrophilic guidewire, were passed through the stricture and introduced into the duodenum or jejunum. The hydrophilic guidewire was then exchanged for a 6F guide sheath, positioned proximal to the stricture. The forceps were introduced into the proximal end of the stricture, with repeated sampling from various locations obtained. Subsequently, brush cytology was performed. The intentional treatment was performed, using either stent deployment or placement of a drainage tube, according to the diagnosis (Figure 2A-2B).

The ERCP procedure was performed using a duodenoscope under intravenous sedation and fluoroscopic monitoring. Once the major duodenal papilla was visible under the endoscope, sphincterotomy of the ampulla of Vater was performed, and the biliary stricture was confirmed via cholangiography. The transpapillary forceps were deployed distal to the biliary stricture. Again, brush cytology was performed after forceps biopsy. The same as PTC, intentional treatment was performed using either stent deployment or drainage tube, based on the diagnosis.

### 2.3 Cytology and pathology

Specimens obtained using brush cytology were fixed onto films, air-dried, and stained using May Grumwald Giemsa for cytological examination. Specimen obtained using forceps were fixed in 10% formalin, as per standard methods. The diagnosis was confirmed by an experienced pathologist who was blinded to the type of procedure (PTC or ERCP).

### 2.4 Statistical analysis

The diagnostic sensitivity, specificity, positive predictive value (PPV), negative predictive value (NPV), and accuracy of brush cytology and forceps biopsy were calculated for PTC and ERCP. Negative outcomes include histological samples classified as suspected malignancy, atypical, or benign, as well as samples insufficient for diagnosis. All data were expressed as mean±standard deviation or percentage. Student *t* test, Pearson Chi-Squared test, and Fisher exact test were performed, as appropriate for the data type and distribution, using SPSS software (SPSS, Inc., Chicago, IL), with a *P*-value <.05 considered statistically significant. The director, Center of Evidence-Based Medicine, the Second Hospital of Shandong University, performed statistical analysis.

## 3 Results

PTC was performed in 38 patients (27 males and 11 females; age, 69.07 ± 9.91 years) and ERCP in 41 (33 males and 8 females; age, 65.07 ± 10.02). The groups did not differ on baseline characteristics (Table 1). Brush cytology and forceps biopsy were successfully performed in all patients. The final diagnosis was confirmed by surgical specimen or clinical/imaging follow-up (range, 6 – 25 months) in patients who did not undergo surgery.

**Figure 3:**
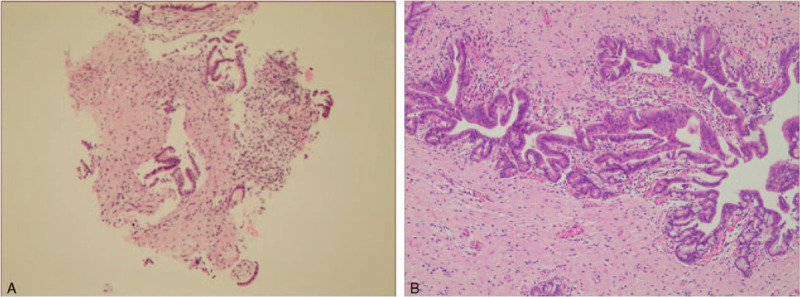
A. cytological and histological samples showed suspected malignancy. 3B. surgical specimen showed cholangitis with no evidence of malignancy.

The sensitivity, specificity, PPV, NPV, and accuracy of brush cytology and forceps biopsy, for PTC and ERCP, are reported in Table 2. In group A (PTC), positive histology/cytology was identified in 26 patients (Figure 2C), with negative results in the other 12, compared to that in 27 patients with positive results and 14 with negative results in group B (ERCP). Based on the reference standard of final diagnosis (group A, surgical confirmation in 15 patients and by follow-up in 23; group B, 21 surgical and 20 by follow-up), a malignant biliary structure was confirmed in 30 patients in group A and 35 in group B. The diagnostic performance of PTC and ERCP was comparable, with a sensitivity, specificity, PPV, NPV, and accuracy of 86.7%, 100%, 100%, 66.7%, and 89.5%, respectively, in group A and 77.1%, 100%, 100%, 42.9%, and 80.4%, respectively, in group B. Five cases with cytological and histological samples showed suspected malignancy, surgical specimen demonstrated no evidence of malignancy (Figure 3A-3B).

**Table 2:**
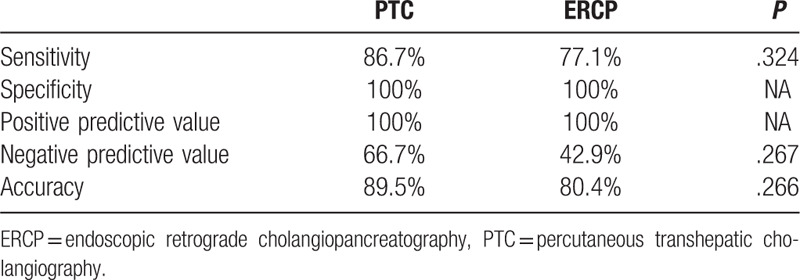
Comparative analysis of diagnostic performance of PTC and ERCP.

In a subset analysis, we evaluated diagnostic sensitivity and accuracy according to the site of the stricture (Table 3). For hilar biliary strictures, the diagnostic sensitivity and accuracy were superior in group A (92.9% and 93.7%, respectively) than in group B (50% and 53.8%, respectively). There was no significant difference in sensitivity and accuracy between group A and group B for lesions located in either the upper or lower segment of the common bile duct. Diagnostic sensitivity for both PTC and ERCP tended to be higher for biliary than for non-biliary carcinoma (Table 4); this finding, however, was not significant.

**Table 3:**
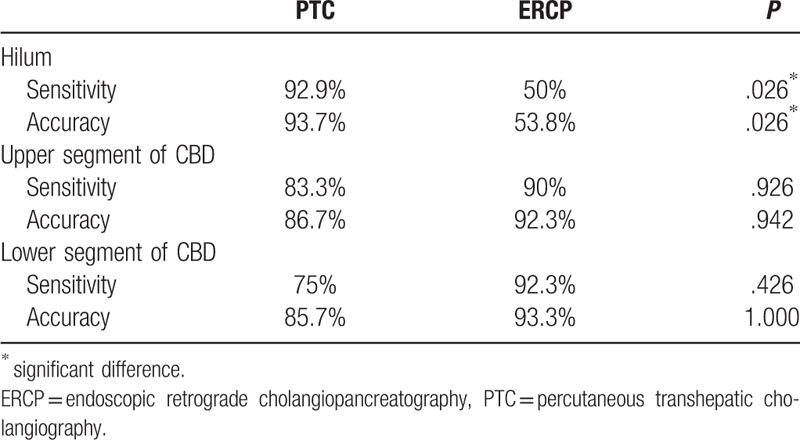
Sensitivity and accuracy of location of the biliary stricture.

**Table 4:**
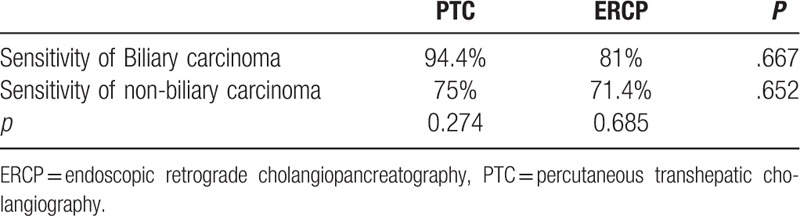
Sensitivity of pathological results.

Procedure-related complications were reported in Table 5. Mild complications occurred in 7 patients in each group: transient haemobilia (4 cases in group A and 5 in group B) and bile leakage (3 cases in group A and 2 in group B). All complications resolved spontaneously within 3 to 5 days. No severe complications requiring reintervention occurred during peri-procedure and follow-up period.

**Table 5:**
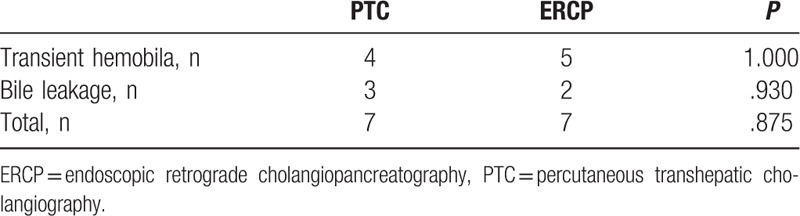
Complications of peri-procedure.

The final negative cases were confirmed by surgical specimen or clinical/imaging follow-up. Surgical specimens with Immunoglobulin G4(IGG4) positive plasma cell infiltrating around the bile ducts were diagnosed as IGG4 related cholangitis. For patients who did not undergo surgery, cholangitis was demonstrated by the imaging features showing no evidence of disease progression and clinic characteristics during follow-up. IGG4 related cholangitis or primary sclerosing cholangitis cannot be exactly distinguished without surgical specimen. More information, such as serum IGG4, should be considered for the diagnosis of IGG4 related cholangitis.

## 4 Discussion

Biliary stricture causes dilation of the bile duct and elevates the level of bilirubin. Noninvasive imaging acts as the first line diagnostic method, providing information of the presence of biliary stricture and the extent of bile duct dilation.^[1]^ However, imaging alone cannot confirm the pathological diagnosis of malignant biliary stricture, with the clinical diagnosis being difficult to make in certain cases. Considering that pre-operative pathological diagnosis in patients with biliary stricture is crucial to inform therapeutic management, especially in cases of malignancy, there is a need to establish the optimal sampling technique to confirm diagnosis.

PTC has been used in the diagnosis and management of biliary stricture since the 1960s.^[3]^ In addition to symptom relief, PTC provides diagnostic information, such as the character and degree of the occlusion. According to previous reports, the sensitivity of cytology sampling during PTC, which includes brush cytology, aspiration sampling and balloon surface sampling, ranges from 47.8% to 61%.^[12–14]^ Forceps biopsy can improve the sensitivity, range from 70% to 88.01%,^[13,15–17]^ with an accuracy for percutaneous transhepatic forceps biopsy (PTFB) of 72% to 91.7%.^[17–19]^ Previous studies have reported PTFB to be more sensitive and accurate than brush cytology in distinguishing malignant biliary stricture.^[14,19]^ Han et al reported a significantly higher true positive rate for biliary carcinoma than for non-biliary carcinoma.^[15]^ Fohlen et al reported greater diagnostic sensitivity for strictures located in the upper (compared to lower) part of the biliary tree.^[17]^ However, Ierardi et al reported a lower diagnostic sensitivity for carcinoma in the hilum and common bile duct than in the common hepatic bile duct and ampulla.^[19]^

In our study, we defined the hilum, including the common hepatic duct, as the extrahepatic bile duct proximal to the orifice of the cystic duct, with the ampullary segment of the common bile duct excluded from the protocol. Based on this definition, we detected higher sensitivity in the hilum than in the common bile duct. Sensitivity was also better for strictures located in the upper than lower segment of the common bile duct. Therefore, specimen sampling of a biliary stricture located proximal to the hilum is simple and effective, yielding a high sensitivity. We do note that our combined use of brush cytology and forceps biopsy yielded higher diagnostic sensitivity and accuracy than previously reported.

PTC for the detection of malignant biliary stricture is easy to perform. Few complications, such as bile leakage, temporary hemobilia, and biloma, have been reported.^[15,17,19]^ Ierardi et al reported that temporary complications, including hemobilia and biloma, occurred in 37.5% of PTC cases, with all complications being related to puncture and not to the endobiliary biopsy.^[19]^ In their study, Ierardi et al confirmed that none of these complications required emergent surgery or blood transfusion for management. Similarly, Fohlen et al reported 4 cases of mild complications in their case series, with 3 (consisting of hemobilia and pneumoperitoneum) resolving spontaneously within 24 hour, and the remaining case (bile leakage) requiring deployment of an external drainage catheter for 1 week.^[17]^ No major adverse events related to forceps biopsy have previously been reported.^[20]^ In our study, PTC with brush cytology and forceps biopsy was performed by, or under supervision of, an experienced interventional radiologist. The procedure was simple and safe to perform, with a few mild adverse events that resolved spontaneously and no serious complications.

More recently, ERCP has quickly been accepted as a feasible diagnostic and therapeutic technique for biliary strictures.^[7]^ ERCP allows tissue sampling and effective management of stones and strictures. Numerous advances in tissue sampling techniques, including bile aspiration, brushing cytology, intraductal biopsy, and fine needle aspiration, can be performed during ERCP.^[9–10,21]^ The sensitivity of ERCP brush cytology for malignant biliary stricture has been reported to range between 41.4% and 62.5%,^[9–10,22]^ with the sensitivity being improved using ERCP forceps biopsy.^[10,21]^ A lower diagnostic sensitivity has been reported for perihilar biliary strictures than for distal biliary strictures.^[10–11,21]^ ERCP forceps biopsy has also been reported to be superior to endoscopic ultrasound-guided fine-needle aspiration for the diagnosis of intraductal lesions.^[22–24]^ However, brush cytology and forceps biopsy at ERCP have limited sensitivity for the diagnosis of malignant biliary strictures^[23]^ and, thus, additional techniques should be used as an alternative to ERCP forceps biopsy for patients with negative pathological findings. The combination of cytological sampling and forceps biopsy might improve the accuracy of pathological diagnosis.^[10]^ In our study, transpapillary sampling with brush cytology and forceps biopsy was performed at ERCP for all patients in group B. Compared to PTC, ERCP provided higher sensitivity and accuracy for strictures located in the lower segment of the common bile duct. As per previous reports, a lower sensitivity was detected for biliary strictures at the hilum, which may be due to the long distance from the papilla of Vater to the site of stricture, with insufficient space for forceps in cases of severe strictures.

Adverse events associated with brush cytology or forceps biopsy are uncommon. One major complication, perforation of the extrahepatic bile duct, treated using nasobiliary drainage, was reported by Yamamoto.^[21]^ Jung et al reported a case of mild pancreatitis (incidence rate of 1.6%) associated to ERCP with papillary biopsy.^[24]^ Dacha et al reported that the overall rate of complication was not significantly different for ERCP performed with or without biopsy (5.6% vs 3.7%). Regardless of forceps biopsy, pancreatic duct manipulation and age were related to adverse events.^[11]^ In our case series of 79 patients, several (n = 14) mild complications occurred, none of which required additional management.

Our results indicate that both PTC and ERCP, using brush cytology and forceps biopsy, provide a sensitive and accurate diagnostic method for suspected malignant biliary strictures. Thus, both PT and ERCP can play an important role in planning the therapeutic strategy for malignant biliary stricture. On direct comparison, PTC (with brush cytology and forceps biopsy) should be considered as a better option than ERCP, providing higher sensitivity and accuracy for stricture locating at the hilum.

## Author contributions

**Conceptualization:** Haiyang Chang, Yongzheng Wang, Yuliang Li.

**Data curation:** Haiyang Chang, Wei Wang.

**Formal analysis:** Haiyang Chang, Yongzheng Wang, Wujie Wang, Wei Wang, Dong Li, Yuliang Li.

**Funding acquisition:** Yuliang Li.

**Investigation:** Haiyang Chang, Bin Liu, Yongzheng Wang, Yuliang Li.

**Methodology:** Haiyang Chang, Bin Liu, Wujie Wang, Wei Wang, Yuliang Li.

**Supervision:** Yongzheng Wang, Yuliang Li.

**Writing – original draft:** Haiyang Chang, Bin Liu, Wei Wang, Dong Li.

**Writing – review & editing:** Haiyang Chang, Bin Liu, Yongzheng Wang, Wujie Wang, Yuliang Li.
